# Supplementary material for: 20 Years of ICF—International Classification of Functioning, Disability and Health: Uses and Applications around the World
Source: Int J Environ Res Public Health. 2022 Sep 8;19(18):11321. doi: 10.3390/ijerph191811321 (PMC9517056; doi:10.3390/ijerph191811321)
Supplement: Supplementary file 1 [file ijerph-19-11321-s001.zip › ijerph-1862112-supplementary.pdf]

SUPPLEMENTARY MATERIALS _Table S1. Main uses table.

World Health Organization - Family of International Classifications Collaborating Centers (WHO-CC FIC) global survey answers by involved countries.

**Country**	**Main uses**
Germany	**Clinical use:** The use of the ICF as basis for instruments for needs assessment is legally backed by the Bundesteilhabegesetz (BTHG), § 118 SGB IX in context of integration assistance for disabled persons, the implementation of ICF will be stepwise introduced in ICF-based needs assessment instruments in German Federal States. The use of ICF is also recommended in the Federal Joint Committee (G-BA) Rehabilitation Guideline. **Health and social policy:** The use of ICF is embedded in legal health and social policies. **Educational use:** Teaching ICF basics and concepts is mandatory and embedded in curricula of several health professions (e.g., specialization of rehabilitation medicine). **Research:** ICF, ICF Core Sets and ICF-based assessments tools have been used in several research projects focusing on a broad range of health conditions and application areas (education setting, rehabilitation, acute treatment) across all age groups.
France	**Health and social policy**: In progress: revising the pricing system of the services provided in the institutions for persons with disabilities (« SERAFIN-PH ») based on ICF domains. **Statistical** (data collection):2008 National Disability-Health survey (activity limitations, participation restrictions, impairments). Next National Disability survey: with WHODAS 2.0 12 items included. **Clinical use**: Checklists / core sets available for Physical and medical rehabilitation, oral health;Orphanet (rare diseases database): ICF domains added. **Educational use: (**about the multidimensional approach of disability)<br>among the mandatory skills for Occupational therapy, Physical therapy;<br>in some university Master degree curricula.

SUPPLEMENTARY MATERIALS _Table S1. Main uses table.
World Health Organization - Family of International Classifications Collaborating Centers (WHO-CC FIC) global survey answers by involved countries.

Denmark	ICF is widley used as a model. **Clinical use:** (Assessment of funtioning after Traumatic brain injury - TBI). National recommendations (August 2020): ICF used as starting point and guiding in TBI: ICF is the reference.
Sweden	**As a framework**: National Board of Health and Welfare - Knowledge support in different areas: *Insurance Medicine Decision Support (IMDS)*; The individual's needs in focus (IBIC) (social care); Municipality health care; Prescription of assistive devices. *General rehabilitation process (ongoing national project):*in different standardized care process for different diagnosis. *Local and regional level.* **Clinical use:***In structured digital documentation (EHR)* **-** Municipality health care (ICF code set); Social care for elderly persons and persons with disability (ICF code set); Used in other areas (i.e rehabilitation) at local and regional level. *In different physician's certificates (ICF implemented):* to assess workability (electronic) and in the Insurance Medicine Decision Support (IMDS); for dental care in case of long-term illness or disability - to assess functioning. **Statistical use:** No collection of functioning data coded with ICF at national level; Support to use ICF at local and regional level for statistics. **Health and social policy:**In 33 Rehab DRG (diagnosis-related groups); In social care for elderly persons and persons with disability; In the Insurance Medicine Decision Support (IMDS);In different certificates for assess functioning. **Educational use:** ICF is used in different educational programmes at universities.

SUPPLEMENTARY MATERIALS _Table S1. Main uses table.

World Health Organization - Family of International Classifications Collaborating Centers (WHO-CC FIC) global survey answers by involved countries.

Italy	ICF is not a mandatory Classification In Italy.<br>**Health and social policy** (in pupils evalution and disability benefits for school children D.Lgs. 66/2017, people with disabilities looking for a job, National disability action plan 1 and 2: WHO DAS 2.0 recognized as base for disability assessment: not implemented yet, invalidy certification in the files of INPS without qualifiers); **Statistica**l (ISTAT; as a descriptor of disability at regional level); **Clinical** (rehabilitation or several individual or institutional initiatives). **Educational use:** (courses in Italian University; training primary and secondary school teachers; research)
The Netherland	**Health and social policy** (Policy design and implementation: e.g. for assistive products); **Educational use:**<br>(Curriculum design: in nursing and allied health professional education, research)<br>**Rehabilitation** (clinical reasoning, registration of data of care, outcome evaluation (rehabilitation professionals, allied health professionals, nurses and occupational health professionals)

Finland	**Educational use:** (basic ICF information and how to use ICF in clinical practice): ICF in student's curriculum to educate all health and social care professionals (doctors, nurse, rehabilitation professionals, etc.) in Universities, Universities in applied sciences and Vocational schools. o ICF education in life-long learning: ICF workshops and training for persons working in clinical practice. Many organizations have educated their professionals to use ICF. **Clinical use:** (practice in several organizations to assess functioning and plan treatment and rehabilitation, e.g (selected examples): Increasing implementation of the ICF in the social and health care sectors all around Finland (e.g., hospitals, health care centers, private rehabilitation units, associations, and other third-sector operators). Implemented nationally in Valteri Centre for Learning and Consulting (Valteri school provides student places is for children in need of special support. It operates under the Finnish National Agency for Education). Implemented in a home care model on the South Carelian Social and Health Care District (Eksote), in services for elderly. This is an integrated service path and rehabilitative operating model, including everyday support. ICF for active use in the rehabilitation of primary health care clinics and early childhood education in the Hospital District of Southwest Finland for children of pre-school age. **Research use:** WHODAS research and in selected examples: iCanFunction (mICF project));Vetovoimala project; KUURA project; INPRO - Interprofessionalism in action!

SUPPLEMENTARY MATERIALS _Table S1. Main uses table.
World Health Organization - Family of International Classifications Collaborating Centers (WHO-CC FIC) global survey answers by involved countries.

Australia	Australia has a broad range of applications of the ICF at the local, state and national levels and the range is expanding. At a national level ICF concepts are used as a basis for (**Haelth and social policy**): disability identification in the Australian Census and in national population surveys run by the Australian Bureau of Statistics (ABS). Long history of national use of ICF concepts and domains: Survey of Ageing, Disability and Carers since 1981; In 2006, disability questions were included in the Census of Population and Housing, also based on ICF concepts; The ABS includes a short 'disability module' in many of its population surveys, enabling thecomparison of people with disability and other Australians; A 'disability flag' has been developed for use in administrative (service) data collections **(Statistical use)** and is based on ICF activity and participation life areas. Australia is transitioning to a new model for providing disability services:the National Disability Insurance Scheme (NDIS) (announced September 2020).<br>At state level, ICF is used as the framework for iCare the NSW Government's insurance and care schemes. The ICF activities and participation life areas are used at the chapter level. **Educational use**: ICF is in the curriculum of allied health professionals in Australia. Increasingly efforts have been made to incorporate ICF the design of state-based collections. ICF is proposed for use in designing resource distribution methods for s**chool education. Research:** ICF has been used to underpin research in speech pathology and physical rehabilitation research and practice. The Australia ICF Interest Group includes researchers, and their work is discussed at the regular meetings. There has been no summarising of the totality of this effort. Other reviews by Australian authors usually cover international research more broadly rather than focussing solely on Australian research.

South Africa	**Educational use**: The conceptual framework of ICF is used in the training of health professions at most universities. **Clinical use**:Conceptual framework used in reporting clinical outcomes and patient assessments at hospitals. **Research**:Research using WHODAS and ICF.

Korea	**Statistical use**: Korea Classification of Functioning, Disability and Health is a modified version of ICF and developed by Statistics Korea. It was enacted and notified as a Korean Standard Health Classification in 2018 and it is recommended to use it for a relevant data collection. However, it has not been implemented yet in any of the national statistics. Questions from the Washington Group used Census 2020 and were adapted into KCF conceptually. Korea Disabled People's Development Institute - Prevalence of disability was investigated using 12 items from MDS-Brief, difficulties in daily living and social participation within persons' environment. It was found to be different when comparing the prevalence rate of the current Korean disability, which is considered to be an impairment-centered assessment and of the ICF concept-based assessment. **Health and social policy:** WHODAS 2.0 was included in the National Health Insurance reimbursement list under the National Health Insurance Act. It is appointed as a symptom assessment tool in neurological functioning examination. Korea Veterans Hospital Service - ICF codes are used for long-term case management and monitoring quality of life. Ninety-eight codes in ICF 2001 were selected, called 'Bohun Classification of Functioning, disability and health'. It is composed of 41 codes for medical impairment and 57 codes for social conditions for needs assessment. 19 ICF codes from social conditions have been adapted in the service system of Veterans Hospital since September, 2020. Seoul Rehabilitation Hospital - ICF codes are used for a team approach in a patient's assessment and treatment plan. A code set is developed based on most commonly used patients' functioning data at the hospital. The code set is implemented in the EMR system used in the hospital. Community Health Centres - Community health centers are running case management programs for Persons with Disability (PwD) discharged from a hospital treatment. Various assessment tools are adapted and used to evaluate functional status of PwD. Development of severe **disabilities assessment (web-based) tool**: 126 ICF codes from activity and participation to assess functioning status in persons with severe disabilities (BusanSocial Welfare Center for Persons with Disability). **Educational use**: A web-based tool has been developed to assess functional status of students with disabilities for the educational support.

Japan	**Clinical use:** In the Japanese insurance system, the preparation of a document named "Rehabilitation Execution Plan" is mandatory for insurance reimbursement for hospital rehabilitation. The document is based on the concept of ICF and is designed to provide information on physical and mental functioning, activities and participation, as well as the contents of rehabilitation for the identified patient issues. However, no ICF codes are used, and the FIM or Barthel Index is used to assess functioning instead.The Ministry of Health, Labor and Welfare has launched the ICF implementation Working Group in the year 2019, to promote the clinical use of ICF and ICD-11 V chapter. The WG is now working on the development of the reference guide for ICF Generic-30 set and ICD-11V chapter, and will finish the first project until 2021 March. A field study is planned within this year. **Educational use:** In Japan, the concept model of the ICF is widely used in education, to understand the relationships between the disease, impairment and activity. However, in most case, the coding is not included in the education.

Russia	**<u>Clinical use</u>:** Disability assessment, Early intervention to children with developmental delays and disorders and their families, Medical rehabilitation of children and adults, Comprehensive rehabilitation, Needs assessment in assisted living (regulated by Giudelines in wich the use of ICF is recommended). **Research sector:** Since 2005 ICF research use have been identified in 422 publications in the Russian Science Library, mainly in the fields of healthcare and rehabilitation, education, and psychology.

Czech Republic	Data sources on functional status in Czechia are several but not based on ICF. **Clinical practice**: use of ICF by some rehabilitation units for measuring disability. **Health and social policy**: some (newer) legislative acts and other regulatory instruments are based on ICF. **Educational use:** university student's curriculum (medicine, physiotherapy, ergotherapy); training provided to rehabilitation and assessment medical doctors, social care professionals and forensic experts; presentations in conferences of professional medical societies and conferences dedicated to classification systems. **Research (selected examples):** EU MHADIE: evaluation of patients after TBI and with MS using ICF checklist and WHODAS. Or Projects on using ICF as a tool for connecting health and social care systems. Or A neuropalliative rehabilitation approach to preserve the quality of life in patients with an advanced stage of selected neurological diseases. Or The CZEch Mental health Study (CZEMS): use of WHODAS to assess disability. Or A study on the usability of the WHODAS 2.0 in geriatric outpatients.

SUPPLEMENTARY MATERIALS _Table S1. Main uses table.
World Health Organization - Family of International Classifications Collaborating Centers (WHO-CC FIC) global survey answers by involved countries.

Canada	**Educational use**: In Canada, the ICF has served as a reference for standardized documentation of health in Occupational Therapy clinical practice, therapy models and derived assessments, such as the Canadian Occupational Performance Measure (COPM), and the Model of Human Occupation Screening Tool. The ICF has been incorporated into the national core curricula for Masters and PhD Physical Therapy and Occupational Therapy programs. **Research**: Canadian researchers in health and the rehabilitation sector in particular, have published numerous articles with a focus on ICF. The Canadian Institute for Health Information (CIHI) conducted a literature review to identify research by Canadian researchers and collaborators which included ICF. Two hundred and twenty-four (224) publications were identified, in the timeframe between 2004 and 2017.

ICF; *International Classifications of Functioning and Health*. FS; Functional Status. NA; *Not Available.*
